# *catena*-Poly[zinc(II)-μ_3_-{hydrogen [1-hydr­oxy-2-(3-pyridinio)ethane-1,1-di­yl]diphospho­nato}]

**DOI:** 10.1107/S1600536809052702

**Published:** 2009-12-12

**Authors:** Xihe Huang, Zhongqian Liu, Changcang Huang, Yubo Wang

**Affiliations:** a Department of Chemistry, Fuzhou University, Fuzhou, Fujian 350002, People’s Republic of China

## Abstract

In the polymeric title compound, [Zn(C_7_H_9_NO_7_P_2_)]_*n*_, the zinc(II) centre displays a tetra­hedral coordination geometry provided by four O atoms from three different phospho­nate groups. The crystal structure consists of ladder chains parallel to the *b* axis built up from vertex-sharing of ZnO_4_ and PO_3_C tetra­hedra. The chains are linked by strong intra- and inter­chain O—H⋯O and N—H⋯O hydrogen bonds, forming a three-dimensional supra­molecular assembly.

## Related literature

For the chemistry and applications of phospho­nate metal derivatives, see: Clearfield (1998 ▶); Cheetham *et al.* (1999 ▶); Maeda (2004 ▶); Gossman *et al.* (2003 ▶); Redman-Furey *et al.* (2005 ▶); Mao *et al.* (2006 ▶); Stahl *et al.* (2006 ▶); Zhu *et al.* (2000 ▶); Burkholder *et al.* (2003 ▶); Bauer *et al.* (2007 ▶); Du *et al.* (2007 ▶). For examples of structure types exhibited by phospho­nate metal derivatives, see: Fu *et al.* (2006 ▶); Yang *et al.* (2007 ▶). For related structures, see: Zhang & Zheng (2008 ▶); Zhang, Gao & Zheng (2007 ▶); Zhang, Bao & Zheng (2007 ▶); Hu *et al.* (2008 ▶).
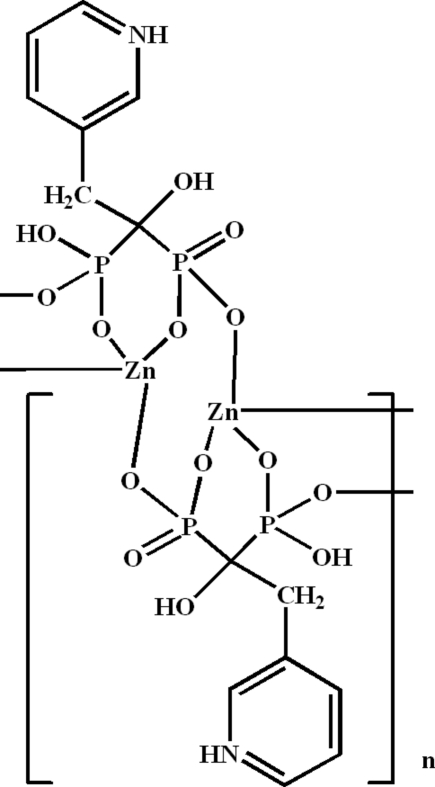


## Experimental

### 

#### Crystal data

[Zn(C_7_H_9_NO_7_P_2_)]
*M* *_r_* = 346.46
Monoclinic, 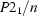

*a* = 13.609 (3) Å
*b* = 5.4809 (11) Å
*c* = 14.818 (3) Å
β = 101.21 (3)°
*V* = 1084.2 (4) Å^3^
*Z* = 4
Mo *K*α radiation
μ = 2.59 mm^−1^
*T* = 293 K
0.20 × 0.12 × 0.08 mm

#### Data collection

Rigaku Mercury CCD area-detector diffractometer
Absorption correction: multi-scan (*RAPID-AUTO*; Rigaku, 1998 ▶) *T*_min_ = 0.626, *T*_max_ = 0.820
8250 measured reflections
2519 independent reflections
2473 reflections with *I* > 2σ(*I*)
*R*_int_ = 0.025

#### Refinement

*R*[*F* ^2^ > 2σ(*F* ^2^)] = 0.026
*wR*(*F* ^2^) = 0.061
*S* = 1.04
2519 reflections
163 parameters
H-atom parameters constrained
Δρ_max_ = 0.35 e Å^−3^
Δρ_min_ = −0.32 e Å^−3^

### 

Data collection: *CrystalClear* (Rigaku, 2002 ▶); cell refinement: *CrystalClear*; data reduction: *CrystalClear*; program(s) used to solve structure: *SHELXS97* (Sheldrick, 2008 ▶); program(s) used to refine structure: *SHELXL97* (Sheldrick, 2008 ▶); molecular graphics: *SHELXTL/PC* (Sheldrick, 2008 ▶); software used to prepare material for publication: *SHELXTL/PC*.

## Supplementary Material

Crystal structure: contains datablocks I, global. DOI: 10.1107/S1600536809052702/rz2390sup1.cif

Structure factors: contains datablocks I. DOI: 10.1107/S1600536809052702/rz2390Isup2.hkl

Additional supplementary materials:  crystallographic information; 3D view; checkCIF report

## Figures and Tables

**Table 1:** Selected bond lengths (Å)

Zn1—O5^i^	1.8791 (16)
Zn1—O2	1.9121 (15)
Zn1—O4^ii^	1.9243 (15)
Zn1—O1^ii^	1.9888 (15)
P1—O2	1.4907 (15)
P1—O1	1.5182 (15)
P1—O3	1.5594 (16)
P1—C1	1.843 (2)
P2—O6	1.4953 (15)
P2—O5	1.5115 (16)
P2—O4	1.5236 (15)
P2—C1	1.851 (2)

Symmetry codes: (i) 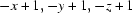; (ii) 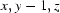.

**Table 2:** Hydrogen-bond geometry (Å, °)

*D*—H⋯*A*	*D*—H	H⋯*A*	*D*⋯*A*	*D*—H⋯*A*
O3—H3⋯O1^iii^	0.82	1.85	2.637 (2)	161
N1—H1⋯O6^iv^	0.86	1.68	2.533 (2)	170
O7—H7⋯O4^ii^	0.82	1.97	2.758 (2)	163

Symmetry codes: (ii) 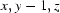; (iii) 
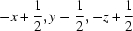
; (iv) 
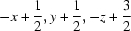
.

**Fig. 1.**
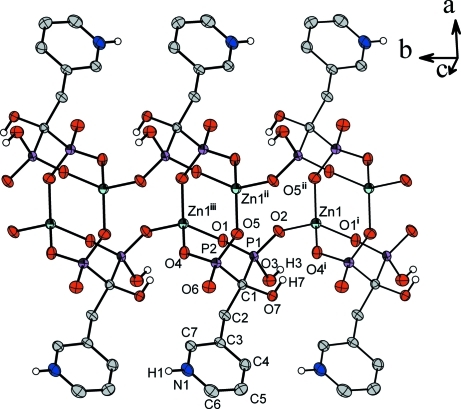
The structure of the title compound, with the atomic numbering scheme of the asymmetric unit and some symmetry-related atoms (50% probability displacement ellipsoids). All H atoms bonded to C atoms are omitted for clarity. Symmetry codes: (i) 1 - x, -y, 1 - z; (ii) x, -1 + y, z; (iii) x, 1 + y, z.

**Fig. 2.**
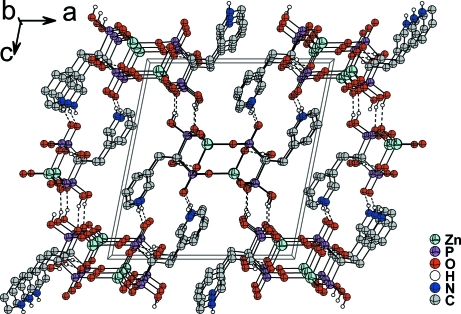
Crystal packing diagram for the title compound. All atoms are shown as isotropic spheres of arbitrary size. H atoms bonded to C atoms are omitted for clarity. The H-bonding interactions are shown as dashed lines.

## supplementary crystallographic information

### Comment

The chemistry of metal phosphonates has gained increasing attention because of its potential applications in catalysis, ion exchange, and magnetic materials (Clearfield, 1998; Cheetham *et al.*, 1999; Maeda, 2004). Many efforts have been devoted to the preparation of metal phosphonate materials with new structure types, especially the open-framework and microporous structures (Clearfield, 1998; Fu *et al.* 2006; Yang *et al.*, 2007). Among these, a promising approach is to modify the organic moieties of the phosphonate ligand RPO_3_^2-^ by other functional groups, such as amino, carboxylate, macrocycle or a second phosphonate group (Zhu *et al.*, 2000; Burkholder *et al.*, 2003; Bauer *et al.*, 2007; Du *et al.*, 2007). Phosphonates based on 1-hydroxyl-1,1-biphosphonic acid, H_2_O_3_PC(OH)(*R*)PO_3_H_2_, such as pamidronate, risedronate, zoledronate and alendronate, are of great research interest because of their applications in therapeutics and as mineral scale inhibitors (Gossman *et al.*, 2003; Redman-Furey *et al.*, 2005; Mao *et al.*, 2006; Stahl *et al.*, 2006). One challenge for studying such materials is that they usually exhibit poor crystallinity, which makes their structural analysis a difficult task. In the case of risedronate acid, (1-hydroxy-2-(3-pyridyl)ethylidene-1,1-diphosphonic acid, abbreviated as H_4_hedp), only five metal complexes have hitherto been structurally characterized, namely, Co_3_(Hhedp)(H_2_O)_4_.H_2_O (Zhang *et al.*, 2008), Co(H_2_hedp)(H_2_O) (Zhang, Gao & Zheng, 2007), Gd(H_2_hedp)(H_3_hedp).2H_2_O (Zhang, Bao & Zheng, 2007), Cd(H_2_hedp)(H_2_O) and Cd_2_Cl(Hhedp)(H_2_O) (Hu *et al.*, 2008). The hedp ligands of these complexs display a variety of coordination modes. We report herein the synthesis and structural studies of a new metal risedronate complex, Zn(H_2_hedp).

The crystal structure of the title complex is built up from one-dimensional covalent zinc phosphonate chains. The asymmetric unit consists of one independent zinc(II) cation and one unique H_2_hedp^2-^ ligand in general position. A detail of the chain structure is illustrated in Fig. 1, showing the coordination geometry of the Zn ion. Every hydrogen phosphonate group of the H_2_hedp^2-^ ligand has two bound oxygen atoms coordinating to the Zn atom and one unbound oxygen atom. The P—O_bound_ bond lengths fall in the range from 1.4907 (15) to 1.5236 (15) Å. The P1—O3 bond length of 1.5594 (16) Å is consistent with the protonation of this unbound oxygen, while the P2—O6 of 1.4953 (15) Å indicates a P=O double bond. The hydroxyl group attached to the C1 atom linking the two phosphorus atoms is uncoordinated, which involves an intramolecular hydrogen bonding interaction with the O4 atom as hydrogen acceptor (Table 2). As shown in Fig. 1, the H_2_hedp^2-^ ligand adopts a (κ1-κ1)-(κ2)-µ3 bridging mode, *i.e.* the H_2_hedp^2-^ ligand coordinates one Zn site in a bidentate fashion forming a Zn1^iii^—O1—P1—C1—P2—O4 six-member chelate ring (symmetry code: (iii) *x*, 1 + *y*, *z*), and two crystallographically equivalent Zn ions in monodentate fashion. The Zn1 atom is tetrahedrally coordinated by four hydrogen phosphonate oxygen atoms from three H_2_hedp^2-^ ligands, with the Zn—O bond lengths in the region of 1.8790 (16)–1.9894 (15) Å, and the O—Zn—O bond angles of 99.06 (6)–117.67 (7)°, respectively (Table 1). Two ZnO_4_ tetrahedra are connected by two P2O_3_C tetrahedra resulting in a Zn_2_P_2_ four-membered ring. These rings are further linked by four PO_3_C tetrahedra through Zn—O bonds, forming a novel one dimensional ladder chain paralleling to the *b* axis. As the best of our knowledge, no examples of this ladder structure had been reported up to date. The chains are cross-linked by strong hydrogen bonds with four adjacent chains to form a three-dimensional supramolecular assembly (Fig. 2). Two interchain hydrogen bonding interactions are observed involving the two unbound hydrogen phosphonate oxygen atoms. The pendant O6 atom as a hydrogen acceptor, is responsible of the first inter-chain H-bond with the protonated pyridyl N atom as donators with the O···N distance of 2.533 (2) Å. The second inter-chain H-bond is constructed from the protonated O3 and O1 atom as hydrogen donators and acceptors, respectively, with O···O distances of 2.637 (2) Å.

### Experimental

NaH_3_hedp.2.5H_2_O (0.1405 g, 0.4 mmol) and ZnO (0.0162 g, 0.2 mmol) were dissolved in 6 ml water. The mixture was placed in a 15-ml Teflon-lined stainless steel vessel and heated at 433 K for 72 h. After slowly cooled to room temperature during 24 h, colourless block crystals of the title complex were collected by filtration, washed with distilled water, and dried in air (yield: 45% on the basis of Zn source).

### Refinement

H atoms bonded to O atoms were located from a difference Fourier map while H atoms attached to C atoms were placed in calculated positions. All H atoms were refined using a riding model approximation, with C—H = 0.93–0.97 Å, O—H = 0.82 Å, and wuth *U*_iso_(H) = 1.2*U*_eq_(C) or 1.5*U*_eq_(O).

### Crystal data

**Table:**

[Zn(C_7_H_9_NO_7_P_2_)]	*F*(000) = 696
*M**_r_* = 346.46	*D*_x_ = 2.123 Mg m^−^^3^
Monoclinic, *P*2_1_/*n*	Mo *K*α radiation, λ = 0.71073 Å
Hall symbol: -P 2yn	Cell parameters from 3898 reflections
*a* = 13.609 (3) Å	θ = 3.1–27.7°
*b* = 5.4809 (11) Å	µ = 2.59 mm^−^^1^
*c* = 14.818 (3) Å	*T* = 293 K
β = 101.21 (3)°	Block, colorless
*V* = 1084.2 (4) Å^3^	0.20 × 0.12 × 0.08 mm
*Z* = 4	

### Data collection

**Table:**

Rigaku Mercury CCD area-detector diffractometer	2519 independent reflections
Radiation source: fine-focus sealed tube	2473 reflections with *I* > 2σ(*I*)
graphite	*R*_int_ = 0.025
ω scans	θ_max_ = 27.7°, θ_min_ = 3.7°
Absorption correction: multi-scan (*RAPID-AUTO*; Rigaku, 1998)	*h* = −17→17
*T*_min_ = 0.626, *T*_max_ = 0.820	*k* = −7→7
8250 measured reflections	*l* = −19→18

### Refinement

**Table:**

Refinement on *F*^2^	Primary atom site location: structure-invariant direct methods
Least-squares matrix: full	Secondary atom site location: difference Fourier map
*R*[*F*^2^ > 2σ(*F*^2^)] = 0.026	Hydrogen site location: mixed
*wR*(*F*^2^) = 0.061	H-atom parameters constrained
*S* = 1.04	*w* = 1/[σ^2^(*F*_o_^2^) + (0.0218*P*)^2^ + 1.6938*P*] where *P* = (*F*_o_^2^ + 2*F*_c_^2^)/3
2519 reflections	(Δ/σ)_max_ = 0.001
163 parameters	Δρ_max_ = 0.35 e Å^−^^3^
0 restraints	Δρ_min_ = −0.32 e Å^−^^3^

### Special details

**Table:**

Geometry. All e.s.d.'s (except the e.s.d. in the dihedral angle between two l.s. planes) are estimated using the full covariance matrix. The cell e.s.d.'s are taken into account individually in the estimation of e.s.d.'s in distances, angles and torsion angles; correlations between e.s.d.'s in cell parameters are only used when they are defined by crystal symmetry. An approximate (isotropic) treatment of cell e.s.d.'s is used for estimating e.s.d.'s involving l.s. planes.
Refinement. Refinement of *F*^2^ against ALL reflections. The weighted *R*-factor *wR* and goodness of fit *S* are based on *F*^2^, conventional *R*-factors *R* are based on *F*, with *F* set to zero for negative *F*^2^. The threshold expression of *F*^2^ > σ(*F*^2^) is used only for calculating *R*-factors(gt) *etc*. and is not relevant to the choice of reflections for refinement. *R*-factors based on *F*^2^ are statistically about twice as large as those based on *F*, and *R*- factors based on ALL data will be even larger.

### Fractional atomic coordinates and isotropic or equivalent isotropic displacement parameters (Å^2^)

**Table:**

	*x*	*y*	*z*	*U*_iso_*/*U*_eq_	
Zn1	0.412237 (17)	0.25347 (4)	0.425508 (16)	0.01698 (8)	
P1	0.29107 (4)	0.76285 (9)	0.37901 (3)	0.01399 (11)	
P2	0.36304 (4)	0.92659 (9)	0.57727 (3)	0.01507 (11)	
O1	0.32521 (11)	1.0088 (3)	0.34936 (9)	0.0191 (3)	
O2	0.36616 (12)	0.5657 (3)	0.37738 (10)	0.0234 (3)	
O3	0.18869 (11)	0.6951 (3)	0.31670 (10)	0.0253 (3)	
H3	0.1983	0.6398	0.2677	0.038*	
O4	0.37845 (11)	1.1832 (3)	0.54302 (10)	0.0201 (3)	
O5	0.44985 (11)	0.7562 (3)	0.57500 (12)	0.0259 (3)	
O6	0.33324 (11)	0.9324 (3)	0.66920 (9)	0.0234 (3)	
O7	0.23742 (11)	0.5508 (3)	0.52251 (10)	0.0211 (3)	
H7	0.2870	0.4642	0.5252	0.032*	
N1	0.10323 (14)	1.1013 (4)	0.71483 (12)	0.0263 (4)	
H1	0.1173	1.2169	0.7545	0.032*	
C1	0.25880 (14)	0.7897 (4)	0.49374 (13)	0.0149 (4)	
C2	0.16165 (15)	0.9394 (4)	0.48646 (13)	0.0190 (4)	
H2A	0.1753	1.1076	0.4727	0.023*	
H2B	0.1121	0.8769	0.4357	0.023*	
C3	0.11892 (14)	0.9324 (4)	0.57271 (13)	0.0179 (4)	
C4	0.06000 (17)	0.7409 (4)	0.59111 (16)	0.0243 (5)	
H4A	0.0452	0.6148	0.5487	0.029*	
C5	0.02268 (19)	0.7328 (4)	0.67077 (17)	0.0295 (5)	
H5A	−0.0182	0.6046	0.6819	0.035*	
C6	0.04682 (18)	0.9168 (5)	0.73327 (16)	0.0301 (5)	
H6A	0.0239	0.9131	0.7884	0.036*	
C7	0.13858 (16)	1.1135 (4)	0.63744 (14)	0.0219 (4)	
H7A	0.1772	1.2468	0.6271	0.026*	

### Atomic displacement parameters (Å^2^)

**Table:**

	*U*^11^	*U*^22^	*U*^33^	*U*^12^	*U*^13^	*U*^23^
Zn1	0.01774 (13)	0.01540 (13)	0.01787 (13)	0.00121 (9)	0.00364 (9)	0.00309 (9)
P1	0.0176 (2)	0.0121 (2)	0.0126 (2)	0.00126 (18)	0.00362 (18)	0.00007 (17)
P2	0.0160 (2)	0.0159 (2)	0.0129 (2)	0.00092 (19)	0.00195 (17)	0.00233 (18)
O1	0.0284 (8)	0.0145 (7)	0.0134 (6)	−0.0008 (6)	0.0019 (5)	0.0020 (5)
O2	0.0305 (8)	0.0180 (7)	0.0246 (7)	0.0093 (6)	0.0122 (6)	0.0046 (6)
O3	0.0231 (8)	0.0350 (9)	0.0175 (7)	−0.0036 (7)	0.0033 (6)	−0.0101 (6)
O4	0.0286 (8)	0.0158 (7)	0.0158 (7)	−0.0013 (6)	0.0044 (6)	0.0013 (6)
O5	0.0165 (7)	0.0231 (8)	0.0373 (9)	0.0042 (6)	0.0033 (6)	0.0036 (7)
O6	0.0264 (8)	0.0304 (8)	0.0135 (6)	0.0012 (7)	0.0040 (6)	0.0025 (6)
O7	0.0232 (7)	0.0145 (7)	0.0280 (8)	0.0001 (6)	0.0108 (6)	0.0037 (6)
N1	0.0304 (10)	0.0289 (10)	0.0197 (9)	0.0020 (8)	0.0050 (7)	−0.0083 (8)
C1	0.0169 (9)	0.0137 (9)	0.0144 (9)	0.0009 (7)	0.0040 (7)	0.0007 (7)
C2	0.0181 (9)	0.0222 (10)	0.0166 (9)	0.0047 (8)	0.0030 (7)	−0.0022 (8)
C3	0.0157 (9)	0.0216 (10)	0.0158 (9)	0.0043 (8)	0.0017 (7)	−0.0026 (8)
C4	0.0226 (10)	0.0243 (11)	0.0253 (11)	−0.0011 (8)	0.0033 (8)	−0.0077 (9)
C5	0.0290 (12)	0.0286 (12)	0.0331 (12)	−0.0038 (9)	0.0116 (10)	0.0025 (10)
C6	0.0342 (12)	0.0360 (13)	0.0222 (10)	0.0064 (10)	0.0106 (9)	0.0013 (10)
C7	0.0207 (10)	0.0222 (10)	0.0234 (10)	0.0011 (8)	0.0059 (8)	−0.0040 (9)

### Geometric parameters (Å, °)

**Table:**

Zn1—O5^i^	1.8791 (16)	O7—H7	0.8201
Zn1—O2	1.9121 (15)	N1—C7	1.329 (3)
Zn1—O4^ii^	1.9243 (15)	N1—C6	1.330 (3)
Zn1—O1^ii^	1.9888 (15)	N1—H1	0.8600
P1—O2	1.4907 (15)	C1—C2	1.542 (3)
P1—O1	1.5182 (15)	C2—C3	1.504 (3)
P1—O3	1.5594 (16)	C2—H2A	0.9700
P1—C1	1.843 (2)	C2—H2B	0.9700
P2—O6	1.4953 (15)	C3—C7	1.370 (3)
P2—O5	1.5115 (16)	C3—C4	1.380 (3)
P2—O4	1.5236 (15)	C4—C5	1.373 (3)
P2—C1	1.851 (2)	C4—H4A	0.9300
O1—Zn1^iii^	1.9888 (15)	C5—C6	1.366 (3)
O3—H3	0.8200	C5—H5A	0.9300
O4—Zn1^iii^	1.9243 (15)	C6—H6A	0.9300
O5—Zn1^i^	1.8791 (16)	C7—H7A	0.9300
O7—C1	1.424 (2)		
			
O5^i^—Zn1—O2	106.21 (7)	O7—C1—C2	106.72 (16)
O5^i^—Zn1—O4^ii^	114.36 (7)	O7—C1—P1	107.57 (13)
O2—Zn1—O4^ii^	113.44 (6)	C2—C1—P1	109.45 (13)
O5^i^—Zn1—O1^ii^	117.63 (7)	O7—C1—P2	110.31 (13)
O2—Zn1—O1^ii^	106.02 (7)	C2—C1—P2	111.50 (14)
O4^ii^—Zn1—O1^ii^	99.08 (6)	P1—C1—P2	111.12 (10)
O2—P1—O1	112.93 (9)	C3—C2—C1	113.26 (16)
O2—P1—O3	110.75 (9)	C3—C2—H2A	108.9
O1—P1—O3	109.18 (9)	C1—C2—H2A	108.9
O2—P1—C1	111.10 (9)	C3—C2—H2B	108.9
O1—P1—C1	109.71 (9)	C1—C2—H2B	108.9
O3—P1—C1	102.68 (9)	H2A—C2—H2B	107.7
O6—P2—O5	112.64 (10)	C7—C3—C4	117.04 (19)
O6—P2—O4	111.29 (9)	C7—C3—C2	121.41 (19)
O5—P2—O4	113.84 (9)	C4—C3—C2	121.53 (19)
O6—P2—C1	108.04 (9)	C5—C4—C3	121.4 (2)
O5—P2—C1	103.62 (9)	C5—C4—H4A	119.3
O4—P2—C1	106.78 (9)	C3—C4—H4A	119.3
P1—O1—Zn1^iii^	128.06 (8)	C6—C5—C4	118.6 (2)
P1—O2—Zn1	144.97 (10)	C6—C5—H5A	120.7
P1—O3—H3	109.5	C4—C5—H5A	120.7
P2—O4—Zn1^iii^	123.97 (9)	N1—C6—C5	119.7 (2)
P2—O5—Zn1^i^	143.43 (10)	N1—C6—H6A	120.2
C1—O7—H7	109.4	C5—C6—H6A	120.2
C7—N1—C6	122.4 (2)	N1—C7—C3	120.9 (2)
C7—N1—H1	118.8	N1—C7—H7A	119.6
C6—N1—H1	118.8	C3—C7—H7A	119.6

Symmetry codes: (i) −*x*+1, −*y*+1, −*z*+1; (ii) *x*, *y*−1, *z*; (iii) *x*, *y*+1, *z*.

### Hydrogen-bond geometry (Å, °)

**Table:**

*D*—H···*A*	*D*—H	H···*A*	*D*···*A*	*D*—H···*A*
O3—H3···O1^iv^	0.82	1.85	2.637 (2)	161
N1—H1···O6^v^	0.86	1.68	2.533 (2)	170
O7—H7···O4^ii^	0.82	1.97	2.758 (2)	163

Symmetry codes: (iv) −*x*+1/2, *y*−1/2, −*z*+1/2; (v) −*x*+1/2, *y*+1/2, −*z*+3/2; (ii) *x*, *y*−1, *z*.
